# Would adding two left atrial piloted images to a cardiac magnetic resonance protocol enable rapid, accurate calculation of left atrial volume? Use of 320 slice cardiac CT as proof of concept.

**DOI:** 10.1186/1532-429X-18-S1-Q51

**Published:** 2016-01-27

**Authors:** Nitesh Nerlerkar, Stuart Moir

**Affiliations:** Monash HEART, Monash Health, Melbourne, VIC Australia

## Background

Left atrial volume (LAV) is an important prognostic predictor in cardiac disease. LAV is not routinely evaluated by cardiac magnetic resonance (CMR) as acquisition of a full volume dataset is time consuming, and previous authors have shown calculation of LAV using the biplane area-length method (BAL) from routinely acquired 4 and 2 chamber views (4CV, 2CV) significantly underestimates true volume. We hypothesized this underestimation was due to standard CMR 4CV and 2CV images (piloted from mid mitral valve to LV apex - LV piloting) foreshortening the atrium, and that additional 4CV and 2CV images piloted from mid mitral valve to the mid posterior wall of the left atrium (LA piloting) would enable rapid, accurate calculation of LAV using BAL.

## Methods

We evaluated 3-D datasets from 44 consecutive patients undergoing retrospective 320 slice cardiac computed tomographic studies. True 3-D left atrial volume (gold standard) was calculated at end systole by a blinded observer excluding pulmonary veins and left atrial appendage. A second blinded observer manipulated images to create standard ‘CMR' 4 and 2 chamber views piloted from mid mitral valve to LV apex (standard LV piloted) enabling measurement of LAV using BAL. The dataset was then manipulated / 're-piloted' from mid mitral valve to the middle of posterior LA (LA piloted) and LAV was re-measured - see figure.

## Results

As previously shown, LAVI calculated with BAL from LV piloted 4CV and 2CV images significantly underestimates true LAV (see table). Mean LAV calculated from LA piloted images was not significantly different from true LA volume and there was a strong correlation between the 2 with narrow confidence intervals.

## Conclusions

Accurate calculation of LAV can be made using BAL method from LA piloted images, and is superior to calculation from standard LV piloted images. Addition of two LA piloted images to a standard CMR protocol may enable rapid and accurate calculation of an important prognostic marker for cardiovascular disease.

**Table 1:** Table 1

Method	Mean ± SD (ml)	95% Confidence Interval	Correlation to 3D LAV (r-value)	Mean difference comparison to 3D-LAV (paired t-test p value)	
3-D LA volume	82 ± 24				
LV piloted LA volume	67 ± 28	(-19,-12)	0.898	<0.001	
LA piloted LA volume	81 ± 27	(-5,1)	0.922	0.27

**Figure 1:**
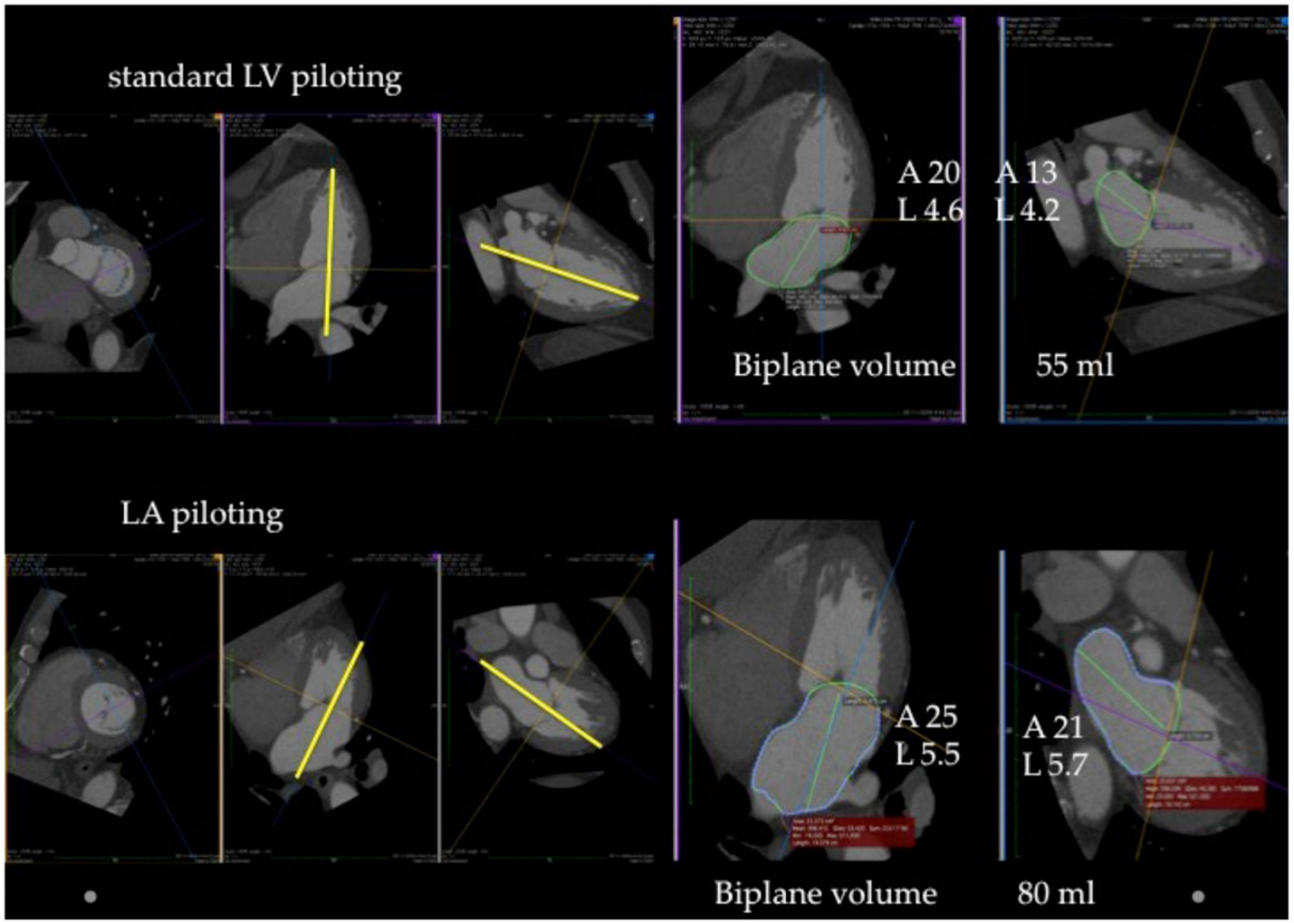
Figure 1

